# Educational Attainment as a Predictor of HIV Testing Uptake Among Women of Child-Bearing Age: Analysis of 2014 Demographic and Health Survey in Zambia

**DOI:** 10.3389/fpubh.2018.00192

**Published:** 2018-08-14

**Authors:** Brian Muyunda, Patrick Musonda, Paul Mee, Jim Todd, Charles Michelo

**Affiliations:** ^1^Department of Epidemiology and Biostatistics, The University of Zambia School of Public Health, Lusaka, Zambia; ^2^Ministry of Health, University Teaching Hospital, Lusaka, Zambia; ^3^Department of Epidemiology and Population Health, Faculty of Epidemiology and Public Health, London School of Hygiene and Tropical Medicine, London, United Kingdom

**Keywords:** PMTCT, education attainment, HIV, DHS, Zambia

## Abstract

**Background:** Globally, an estimated 150,000 children were newly infected with HIV in 2015, over 90% of them in Sub-Saharan Africa. In Zambia, ~500,000 babies are born to HIV positive mothers every year, and without intervention 40,000 of them would acquire the infection. Studies have shown a strong association between education and HIV prevalence, but in Zambia, this association has not been demonstrated. There is little published information on the association between educational attainment and HIV testing uptake among pregnant women, which is fundamental in understanding the mother to child transmission of HIV. This study investigated whether educational attainment was associated with uptake of HIV testing among women of reproductive age in Zambia.

**Methods:** Data were taken from Zambia Demographic and Health Survey in 2014 (ZDHS14). The analysis consisted of all women aged 15–49 years, who responded to the question on HIV testing in the ZDHS. Multivariable logistic regression was used to determine whether educational attainment was associated with uptake of HIV testing among women of reproductive age in Zambia.

**Results:** Educational attainment was strongly associated with HIV testing among 15,388 women of child bearing age [AOR 3.8, 95% CI 1.7–8.2; *p* = 0.001]. HIV testing differed greatly by socioeconomic social status with an increased uptake among women with higher wealth index [AOR 4.4, 95% CI 1.9–9.9; *p* = 0.001]. Additionally, HIV testing was observed to be higher among the older women 25–34 years compared to the young women 15–19 years [AOR 2.3, 95% CI 1.3–4.3; *p* = 0.007].

**Conclusions:** This study revealed educational attainment to be a strong predictor of HIV testing among women of child bearing age in this population. High HIV testing uptake among educated pregnant women indicated that low-educated women may not fully realize the benefits of testing for HIV. Therefore, strengthening HIV testing in rural health facilities and providing initiatives to overcome barriers to testing among women with no formal education may help reduce vertical transmission of HIV.

## Background

Vertical transmission of HIV from mother to child remains the primary mode of infection in children. Globally, an estimated 150,000 children were newly infected with HIV in 2015, over 90% of them in Sub-Saharan Africa through mother to child transmission of HIV (MTCT) (1). In the three years between 2012 and 2014, more than 240,000 new-borns were newly infected with HIV, 90% of them in Sub-Saharan Africa (2, 3). In resource constrained countries, approximately one third of HIV infected infants die before reaching one year and more than half die before reaching two years of age (4).

Prevention of mother to child transmission (PMTCT) programs have been identified as a primary strategy to decrease vertical transmission of HIV (5–10). Making PMTCT services accessible is essential for the eventual elimination of infant HIV transmission in Sub-Saharan Africa. However, a number of studies have shown that PMTCT services have poor coverage and are not easily accessible to all pregnant women. A study from Ethiopia in 2011 showed that, although voluntary testing was provided freely, there were progressive losses to follow-up of 55, 68, and 70% of HIV-positive mothers during the antenatal period, delivery, and first postnatal visits, respectively and many deliveries occurred at peripheral sites where PMTCT was not available (11).

Studies have also shown that both social-demographic factors and health system factors have an influence on the accessibility and uptake of HIV services in PMTCT. The socio-demographic factors include woman's educational status (12–16); late infant diagnosis and adherence to treatment (17); failure to deliver at a health facility (18) as well as stigmatization and discrimination (19–21). In Ethiopia, mothers with a secondary level education were found to be more knowledgeable about mother-to-child transmission and PMTCT, although only a very small proportion knew that elective cesarean section could be used as a method to prevent MTCT. The study also showed that poor socioeconomic status and fear of HIV related stigma influenced the choice of delivery location, irrespective of maternal educational level (11). Many of these HIV positive women fear that if they delivered at the health facility, their friends and relatives would know their HIV status and so home delivery was considered a solution to stigmatization. Another study on utilization and acceptability of PMTCT services showed that many women declined HIV testing in ANC due to poor understanding of HIV testing (22–28).

Zambia lies in the HIV contiguous belt stretching from Uganda in East Africa, southwards to Botswana and South Africa (29–36). The PMTCT programme in Zambia started around 1999. Annually, ~500,000 babies are born in Zambia (37), with an antenatal HIV prevalence of 16.4% in 2008. This means that each year 80,000 infants are at risk of getting infected with HIV/AIDS from their mothers [(38, 39); WHO -PMTCT vision 2010-2015]. According to the Zambia Demographic and Health Survey (ZDHS), Zambia records an infant mortality of 45 per 1,000 live-births and an under-five mortality of 75 per 1,000 live-births and about 10% of all new HIV infections are attributable to MTCT [ZDHS 2013-2014;(4)].

Prevention of mother to child transmission of HIV is a key intervention for pregnant women. All women attending ANC are counseled and tested for HIV, and those positive are initiated on lifelong ART in order to reduce the probability of transmitting HIV to their children. HIV positive pregnant women are given additional interventions and encouraged to deliver at a health facility where prophylactic ARV drugs can be given to the new-born babies. The ZDHS report shows that out of 16,411 women who gave birth, 88% received HIV counseling during Antenatal care visits (ZDHS 2014 Report). Among the topics covered during counseling were: the risk of babies getting AIDS virus from mother; preventing transmission of the virus and the reasons for getting tested for the virus. All women who were tested for HIV received the test results and post-test counseling. Additionally, 91% of the women had an HIV test either during antenatal care or during labor for their most recent birth and received the test results. In general, the percentage of women who have been counseled, tested and received the test results increased with increasing education and wealth index. Furthermore, increasing the percentage of births delivered in the health facilities is an important factor in reducing deaths arising from complications of pregnancy. The percentage distribution of livebirths in the five years preceding the survey by place of delivery showed that 67% of births took place in a health facility with 31% at home. Delivery at a health facility was strongly associated with mothers education and wealth (ZDHS 2014 Report).

A systematic review on educational attainment and HIV 1 infection in developing countries showed that earlier studies (before 1996) tended to find either no association with education level or a higher risk of HIV infection among the most educated (40). On the other hand, a proportion of studies conducted from 1996 onwards identified a lower risk of infection among the most educated [(40–44); ZDHS Report, 2014]. However, there is little published information on the association between educational attainment and HIV testing uptake among women which is fundamental to understanding the differentials in HIV transmission. Therefore, in this study we investigate the association between educational attainment and HIV testing uptake among women of reproductive age in Zambia.

## Methods

### The 2014 ZDHS design

#### Population and sampling procedures

The data analyses are from the Zambia Demographic and Health Survey conducted in 2014 (45). The survey selected a representative sample of 16,411 women aged 15–49 years using stratified random-cluster sampling method. The detailed methods and key findings of the ZDHS 2014 have been reported elsewhere (ZDHS 2014 Report; Census 2010 Report).

From the 16,411 women captured in the survey, 15,388 women who responded to the question on HIV testing in the ZDHS comprised the de facto eligible sample. The extracted data on these women included information on education status, socio-demographic characteristics, and history of antenatal care for the most recent birth within a 5-year period preceding the survey, including HIV testing uptake at ANC attendance. All questions from the 2014 Zambia DHS that were used in the analysis are shown in Table 1.

**Table 1:** All extracted questions from the Women's Questionnaire, 2014 Zambia DHS that were used in the analysis.

**S/N**	**Variable name**	**Variable number**	**Variable type**
1	Cluster Number	V001	Variables used in calculating a Weighted analysis
2	Household Number	V002	
3	Respondents Line Number	V003	
4	Women's Individual Sample weight	V005	
5	Primary Sampling Unit (PSU)	V021	
6	Sample Strata for sampling error	V022	
7	Age (continuous)	V012	Independent variables
8	Type of place of residence	V102	
9	Highest educational level	V106	
10	Wealth Index	V190	
11	Total children ever born/number of living children	V201	
12	Currently Pregnant	V213	
13	Wanted Pregnancy when became pregnant	M10_1	
14	Place of delivery	M15_1	
15	Current Marital status	V501	
16	Husband/Partner education level	V701	
17	Husband/Partner age	V730	
18	Received result from last HIV test	V828	
19	During antenatal visit talked about: HIV transmitted mother to child	V838a	
20	Offered HIV test as part of antenatal visit	V839	
21	Tested for HIV as part of antenatal visit	V855	
22	Received counseling after tested for AIDS	S413a_1	
23	Ever been tested for HIV	V781	Dependent variable

### Statistical analysis

STATA software version 14.0 (College station, Texas, USA) was used for all analyses. Multivariable logistic regression incorporating the ZDHS survey weights were performed, to obtain odds ratios (OR) and 95% confidence intervals (95% CI). Estimates were adjusted for age and other confounders to assess the contrast in uptake of HIV testing between women with lower and higher education. The likelihood ratio test was used to assess the significance of risk factors and interactions. We assessed interactions between wealth index and educational level; age and wealth index; age and educational level as well as place of delivery and educational level. We also assessed collinearity and correlation between the variables “Whether ANC included HIV test or not?” and “ANC covered PMTCT.” In assessing the effect of education on HIV testing further, we adjusted for ever had a child (using the proxy question “Total children ever born to a woman,”) and “Currently pregnant.”

All variables that were statistically significant at 0.05 level in the bivariate analysis were included in the multivariable regression analysis. The variables in the multiple logistic regression model were age, education (no education, primary, and secondary or higher), residence (urban or rural), marital status (Never in union, married, divorced/separated, widowed), wealth index used as a proxy for social economic status (rich, medium, poor), partner age, and partner educational level (no education, primary and secondary, or higher) as well as place of delivery information (home, Government hospital, or private health facility/other).

### Ethics

The Survey protocol obtained clearance from Tropical Diseases Research Centre (TDRC) in Zambia and the US Centres for Disease Control and Prevention (CDC) Atlanta Research Ethics Review Board (ZDHS 2014 Report). Participation in the survey was voluntary and written informed consent was obtained. The ZDHS data were anonymised and made available for public use, and reanalysis of the data did not interfere with participant's privacy and posed minimal to no risk. For this analysis, a waiver was obtained from the University of Zambia Biomedical Research Ethics Committee (UNZABREC). (Ref. no. 010-04-16).

## Results

### Participation and distribution

Overall, our target population for this study were women who had ever tested for HIV. Our aim was to understand the general HIV testing uptake among women of child-bearing age and also among women who had attended ANC. The prevalence forever tested for HIV was 80% and the other 20% most likely included young women still in school and had never had sex. From the eligible 16,411 women aged 15–49 years in the survey, 15,388 women had data on their HIV testing. Of these 15,388 women, the median age was 27 years (IQR = 23 to 33 years) and mean age was 28 years (SD ± 9.4) with 4,901 (32%) of women in the age range 25-34 years. Distribution by residence showed that 7,316 (47%) women were living in urban areas and 9,101 (59%) were married (Table 2).

**Table 2:** Social Demographic Characteristics of 15,388 women aged 15–49 years in Zambia who had data on HIV testing.

**Characteristics**	**Frequency**	**(Percent)**
**AGE GROUP (IN YEARS)**		
15–19	3,485	22.7
20–24	2,881	18.7
25–34	4,880	31.7
35–49	4,142	26.9
Total	15388	100.0
**RESIDENCE**		
Urban	7,299	47.4
Rural	8,089	52.6
Total	15388	100.0
**EDUCATIONAL LEVEL**		
No education	1,253	8.2
Primary	7,264	47.2
Secondary or higher	6,862	44.6
Total	15388	100.0
**MARITAL STATUS**		
Not Married	4,453	28.9
Married/Living together	9,067	58.9
Widowed/Divorced	1,548	10.1
Separated	320	2.1
Total	15388	100.0
**WEALTH INDEX (QUINTILES)**		
Poor	5,528	35.9
Medium	3,317	21.6
Rich	6,543	42.5
Total	15388	100.0
**PARTNER EDUCATION LEVEL**		
No education	930	8.5
Primary	4,047	37.2
Secondary or higher	5,908	54.3
Total	10885^*^	100.0

* *The total sample size reduce (from n = 15,388) because some variables had missing values and were excluded in the analysis*.

Distribution of educational attainment by residence showed that of the total 6,768 women who attained secondary education or higher as their highest educational level, 4,471 (66%) women were living in the urban areas compared to 2,297 (34%) women in the rural areas (*P* < 0.001). Of the 10,926 partners to the women, 45% (4,904) reported secondary school as their highest level of education attained. Furthermore, 7,264 (47%) women reported primary school as their highest educational level attained while 1,253 (8%) women had no formal education (Table 2).

### HIV test uptake by educational attainment

Out of the total of 6,768 women who reported secondary education or higher, 5,426 (80%) had tested for HIV compared to 1,342 (20%) women who had not (*P* < 0.001). Similarly, 4,707 (94%) women whose partners had attained secondary education or higher had tested for HIV compared to 300 (6%) women who had not tested (*P* < 0.001) (Table 3). Uptake of HIV testing differed greatly by educational level of woman with an increased uptake observed among women of secondary education or higher [AOR 3.8, 95% CI 1.7–8.2; *p* = 0.001] (Table **5**).

**Table 3:** Background Characteristics of 15,388 Women aged 15–49 years on HIV Testing, in Zambia with data on HIV testing, a bivariate analysis using Pearson Chi squared test.

***N* = 15,388**	**Ever Tested for HIV**				
	**No**		**Yes**		***P*–Value**
**Characteristics**	***n***	**(%)**	***n***	**(%)**	
**Educational Level**					<0.001^*^
No Education	288	022.9	969	77.1	
Primary level	1,327	018.3	5,927	81.7	
Secondary level	1,342	019.8	5,426	80.2	
**Age group (in Years)**					** <0.001**^a^
15–19	1,723	50.6	1,681	49.4	
20–24	318	11.2	2,530	88.8	
25–34	334	6.8	4,561	93.2	
35–49	582	14	3,560	86	
**Residence**					**0.023**
Urban	1,258	18.1	5,694	81.9	
Rural	1,699	20.4	6,639	79.6	
**Marital Status**					**<0.001**
Not Married	1,941	45.7	2,310	54.3	
Married/Living together	817	8.9	8,406	91.1	
Widowed	174	11.7	1,316	88.3	
Divorced	25	7.6	301	92.4	
**Wealth Index (Quintiles)**					**0.022**
Poor	1,115	20.7	4,279	79.3	
Medium	504	17.3	2,409	82.7	
Rich	1,338	19.2	5,645	80.8	
**Place of Delivery**					**<0.001**
Home	296	12.4	2,091	87.6	
Government Facility	187	3.2	5,656	96.8	
Other	16	3.3	498	96.7	
**Partner Educational level**					**<0.001**
No Education	158	16.0	774	83.0	
Primary level	505	12.2	3,625	87.8	
Secondary or higher	300	6.0	4,707	94.0	

* P <0.05 was considered statistically significant at 95% confidence level.

a *Pearson Chi2 test was used to obtain p–values*.

### Other determinants of HIV testing uptake among women 15–49 years

HIV testing was observed to be higher among the older women (25–34 years) compared to the younger women (15–19 years) [AOR 2.3, 95% CI 1.3–4.3; *p* = 0.007]. Furthermore, rural women had lower odds of testing for HIV compared to those in urban areas [OR 0.8, 95% CI 0.7–0.9; *p* = 0.02], though this was not statistically significant in the multiple regression. High HIV uptake was also observed among women who delivered from the health facility whether Government or private compared to those who delivered from home [AOR 3.0, 95% CI 1.3–6.8; *p* = 0.013] (Table 5). The assessed interactions between wealth index and educational level; age and wealth index; age and educational level as well as place of delivery and educational level were considered not important because they were not statistically significant. The collinearity assessment between the independent variables ANC included HIV and ANC covered PMTCT showed that both the regression analysis tolerance (0.893) and the variance inflated factor (1.12) were close to 1 implying that the variables were either uncorrelated or had a weak correlation.

**Table 4:** Characteristics of 6,506 women who reported tested for HIV during ANC clinic in the last 5 years in Zambia, a bivariate analysis using Pearson Chi squared test.

***N* = 6,506**	**Tested for HIV in ANC in the last 5 years**						
	**No**			**Yes**			***P*–Value**
**Characteristics**	***n***	**(%)**	**95% CI**	***n***	**(%)**	**95% CI**	
**Educational Level**							<0.001^*^
No education	67	10.3	[7.8–13.3]	586	89.7	[86.7–92.2]	
Primary	198	5.6	[4.5–7.1]	3,332	94.4	[92.9–95.5]	
Secondary or higher	53	2.3	[1.7–3.2]	2,215	97.7	[96.8–98.3]	
**Age group (in Years)**							0.275^a^
15–19	44	5.7	[4.0, 8.1]	719	94.3	[91.9, 95.8]	
20–24	73	4.4	[3.3, 6.3]	1,564	95.6	[93.7, 96.7]	
25–34	130	4.6	[3.7, 5.8]	2,713	95.4	[94.2, 96.3]	
35–49	72	5.9	[4.5, 7.7]	1,142	94.1	[92.3, 95.5]	
**Residence**							**<0.001**
urban	35	1.6	[1.1, 2.3]	2,174	98.4	[97.7, 98.9]	
rural	283	6.7	[5.6, 8.2]	3,965	93.3	[91.8, 94.4]	
**Marital Status**						0.456
Not Married	24	3.6	[2.3, 5.6]	638	96.4	[94.4, 97.7]	
Married/Living together	261	5.1	[4.2, 6.1]	4,954	94.9	[93.9, 95.8]	
Widowed	23	5.6	[3.5, 8.6]	389	94.4	[91.4, 96.5]	
**Wealth Index (Quintiles)**							**<0.001**
Poor	224	7.6	[q]	2,785	92.4	[q]	
Medium	55	4.1	[3.0, 5.6]	1,304	95.9	[94.4, 97.0]	
Rich	38	1.8	[1.2, 2.7]	2,050	98.2	[97.3, 98.8]	
**Place of Delivery**							**<0.001**
Home	169	9.9	[q]	1,548	90.1	[q]	
Government Facility	137	3.2	[3.0, 5.6]	4,184	96.8	[94.4, 97.0]	
Other	12	1.8	[1.2, 2.7]	406	98.2	[97.3, 98.8]	
**ANC Covered PMTCT**							**<0.001**
No	125	31.0	[25.1, 37.6]	277	69.0	[62.4, 74.9]	
Yes	197	3.3	[2.6, 3.9]	5,862	96.7	[96.0, 97.4]	
**Partner Educational Level**							**<0.001**
No education	37	7.3	[5.0–10.5]	467	92.7	[89.5–94.9]	
Primary	174	7.3	[5.8–9.0]	2,214	92.7	[90.9–94.2]	
Secondary or higher	83	2.9	[2.1–3.9]	2,801	97.1	[96.1–97.9]	

* P <0.05 was considered statistically significant at 95% confidence level.

a *Pearson Chi2 test was used to obtain all p-values*.

*^q^ Confidence intervals could not be obtained*.

**Table 5:** Key Predictors of HIV Testing Among women aged 15–49 years who participated in the Demographic and Health Survey 2014- from Multivariable Logistic Regression Analysis.

***N* = 15,388**		**Crude**			**Adjusted**		
**Characteristic**	**Category**	**OR**	**95% CI**	***P*****-Value**	**AOR**	**95% CI**	***P*****-Value**
**EDUCATIONAL LEVEL**							
	No Education	Ref			Ref		
	Primary	1.3	[1.1–1.6]	**0.003**	1.4	[0.7–2.3]	0.406^a^
	Secondary/Higher	1.2	[1.0–1.5]	**0.011**	3.8	[1.7–8.2]	0.001^*^
**AGE GROUP (IN YEARS)**							
	15–19	Ref			Ref		
	20–24	8.2	[6.9–9.7]	**<0.001**	2.2	[1.1–4.0]	**0.017**
	25–34	13.9	[11.6–16.8]	**<0.001**	2.3	[1.3–4.3]	**0.007**
	35– 49	6.2	[5.4 – 7.3]	**<0.001**	1.9	[0.9–3.6]	0.069
**MARITAL STATUS**							
	Single	Ref			[b]		
	Married	8.6	[7.5–10.0]	**<0.001**			
	Widowed/Divorced	6.3	[5.2–7.8]	**<0.001**			
	Separated	10.2	[6.1–16.9]	**<0.001**			
**WEALTH INDEX**							
	Poor Class	Ref			Ref		
	Middle Class	1.2	[1.1–1.4]	**0.001**	1.4	[0.8–2.4]	0.213
	Rich Class	1.1	[0.9–1.3]	0.162	4.4	[1.9–9.9]	**<0.001**
**RESIDENCE**							
	Urban	Ref			[b]		
	Rural	0.8	[0.7–0.9]	**0.023**			
**PLACE OF DELIVERY**							
	Home	Ref			Ref		
	Government Facility	4.3	[3.3–5.5]	**<0.001**	2.0	[1.2–2.9]	**0.007**
	Private Facility/Other	4.2	[2.4–7.8]	**<0.001**	2.9	[1.3–6.8]	**0.013**
**ANC COVERED HIV PMTCT**							
	No	Ref			[b]		
	Yes	11.2	[7.9–15.9]	**<0.001**			
**ANC INCLUDED HIV TEST**							
	Yes	Ref			Ref		
	No	245.2	[159.2–377.7]	**<0.001**	198.8	[127.3–310.3]	**<0.001**
**PARTNER EDUCATIONAL LEVEL**							
	No Education	Ref			[b]		
	Primary	1.5	[1.1–1.9]	**0.003**			
	Secondary or higher	3.3	[2.4–4.2]	**<0.001**			

* P <0.05 was considered statistically significant at 95% confidence level.

a *Pearson Chi–squared test was used to obtain all p-values*.

*^b^ Marital status, residence, ANC covered PMTCT and partner education were not statistically significant after adjustment in the stepwise regression, hence AOR not shown in the Table 4*.

*Bold Values indicate all P-values that are <0.05 and statistically significant*.

## Discussion

The findings have revealed a strong association between educational attainment and uptake of HIV testing. In the univariate analysis, the association between HIV testing and educational attainment was not strong. However, after adjusting for confounding effect of age, wealth index and place of delivery, the association between uptake of HIV testing and educational attainment strengthened. We observed an overall increase in the reported uptake of HIV testing among ANC attenders which builds on the finding from an earlier study in which educational attainment was associated with optimal ANC attendance which includes HIV testing (46). This finding suggests that educated women may be more knowledgeable about MTCT of HIV and understand the benefits of testing for HIV during pregnancy. This finding reaffirms the results from other Sub-Saharan African countries showing that mothers with a secondary educational level or above are likely to be more knowledgeable about mother-to-child transmission (11, 12). Furthermore, studies on utilization and acceptability of PMTCT services showed that many women decline HIV testing in ANC due to poor understanding of PMTCT interventions (11, 19, 22). Improving the education of female children is very important if we are to increase PMTCT utilization. The Zambian Government has in the recent past put up measures to enhance girl's retention rates in school. One such measure is to allow girls who drop out of school due to pregnancy return and continue their education after delivery. Additionally, village chiefs and headmen have been involved in the fight against early marriages, and encouraging girls to continue at school.

Facility delivery is one of the key components in a successful PMTCT programme. Women who delivered at health facilities were more likely to undergo HIV testing compared to those that delivered from home. However, consistently low facility delivery is associated with lower HIV testing uptake among ANC women thereby exposing their unborn babies to vertical transmission. One study observed similar results that failure to deliver from health facility reduces chances of women to utilize HIV testing services (18).

Over the past decades, a series of Population-based surveys have been conducted in Zambia from 1992 through 2010 (ZDHS Report 1992-2014; ZSBS Report 2009; Census Report 2010). The data showed extremely high HIV prevalence levels among child bearing women. HIV infection was found to rise sharply with increase in educational attainment (29). Later other study findings in Zambia and Uganda suggested a paradigm shift in the association between educational attainment and HIV infection indicating that high educational attainment was actually protective and that the previous finding was just a sign of an on-going process of significant behavioral change (41, 42, 44). Understanding the dynamics of HIV testing uptake in association with educational attainment is crucial because differential testing patterns among women with varied educational levels may bias the findings. This study also revealed differential patterns of HIV testing associated with socio-economic status of woman. In comparing women of low economic status and those with a high one, the latter were more likely to have an HIV test.

Selection biases such as non-participation by not attending ANC and absence during the ZDHS survey (due to school, or traveling) could have possibly affected our findings and estimates. However, the magnitude of this effect can only be assessed through other studies with a similar design. Participation in this survey had a response rate of about 98% implying that the minimal absence was because of non-availability during survey. Therefore, we are very confident that the non-participation was very minimal and not important in altering our findings.

As in most of secondary analysis-based studies, the data were not tailored with this objective as a focus and as such predictor measures may not capture exactly initial measures intended and we tried to controlled for the effect of confounding through stratification during the analysis process. We further adjusted for ever had a child (used a proxy question “Total children ever born to a woman,” and “Currently pregnant,” but this did not have effect on the association of education and HIV testing. It may not be possible to conclusively control for all confounding and interactions in these findings due to uncontrolled confounding effect that may result from other forms of non-participation bias such as misclassification of missing values which is a threat to most data. In this analysis taking into account all these limitations, we still believe their effect is minimal and unimportant in explaining our findings. Hence these findings may help programme implementers and policy makers in channeling limited resources and interventions in particular areas of real need for better maternal and new-born health outcomes.

## Conclusions

The study revealed that there is a strong association between educational attainment and HIV testing. We have demonstrated that HIV testing uptake is high among educated urban women. Early marriages and high drop-out rates among rural girls at primary and secondary levels could be a possible explanation for this disparity. Therefore, addressing the educational needs of rural and low-educated women is crucial if they are to benefit fully from the PMTCT interventions.

## Recommendations

Considering that educational attainment can be linked to increased HIV test uptake, supporting existing Government measures to retain girls in school and strengthen educational structures in order to improve access to universal secondary education among predominantly lower educated groups and rural young women is a key requirement in addressing barriers to increased uptake of PMTCT. Strengthening HIV testing in rural health facilities, encouraging women to deliver in facilities and provide initiatives that seek to overcome barriers to testing among those with no formal education are some of the ways that can help improve maternal and new-born health.

## Availability of data and materials

The data that support the findings of this study are available from the Zambia Central Statistical Office and Ministry of Health. These datasets can be obtained on request from the Zambia Central Statistical Office and also available through the website from the Measure Consortium (https://www.dhsprogram.com/).

## Authors contributions

BM conceived the study ideas, design, analyzed data and wrote the draft manuscript. CM participated in the designing of the concept, methods, analysis, and edited the manuscript. PMe contributed to the analysis and edited the manuscript and made contributions. JT participated in the study design, edited the manuscript, and contributed to the final analysis. PMu edited the manuscript and contributed to the analysis.

### Conflict of interest statement

The authors declare that the research was conducted in the absence of any commercial or financial relationships that could be construed as a potential conflict of interest.
